# Downstream Change of the Primary Endpoint in the ISCHEMIA Trial: the Elephant in the Room

**DOI:** 10.5935/abc.20180145

**Published:** 2018-08

**Authors:** Luis Cláudio Lemos Correia, Anis Rassi Junior

**Affiliations:** 1 Escola Bahiana de Medicina e Saúde Pública - Hospital São Rafael, Fundação Monte Tabor, Salvador, BA – Brazil; 2 Hospital do Coração - Anis Rassi, Goiânia, GO – Brazil

**Keywords:** Myocardial Ischemia/physiopathology, Cardiac Catheterization, Myocardial Revascularization, Treatment Outcom, Evaluation Studies

The ongoing ISCHEMIA trial (International Study of Comparative Health Effectiveness with Medical and Invasive Approaches, NCT01471522),^1^^,^^2^ aims to overcome the limitations of previous trials to identify the best management strategy for patients with stable ischemic heart disease (SIHD). Percutaneous coronary intervention (PCI), the most common form of coronary revascularization, and coronary artery bypass grafting (CABG) have been used successfully to improve angina symptoms and quality of life in patients with severe coronary obstructions.

The findings of recent trials such as COURAGE (Clinical Outcomes Utilizing Revascularization and Aggressive Drug Evaluation), BARI 2D (Bypass Angioplasty Revascularization Investigation 2 Diabetes), and FAME 2 (Fractional Flow Reserve versus Angiography for Multivessel Evaluation) fail to show any difference in mortality or myocardial infarction (MI) between patients with SIHD who were treated invasively compared with those who were treated with optimal medical therapy (OMT). However, these trials had a substantial proportion of patients with no significant myocardial ischemia, potentially underestimating the beneficial effect of revascularization. Therefore, the ISCHEMIA trial was set to resolve this issue by including only patients with moderate-to-severe ischemia inducible at stress imaging.

The primary aim of the ISCHEMIA^1^^,^^2^ trial is to test the hypothesis that the use of a routine invasive strategy with cardiac catheterization followed by revascularization (PCI or CABG) plus OMT is superior to a conservative strategy of OMT, with cardiac catheterization and revascularization reserved for those who fail OMT. ISCHEMIA is a major study funded by the National Institutes of Health and is the largest clinical trial comparing alternative treatment strategies in patients with SIHD. With an average follow-up period estimated to be about 3.5 years (minimum of 12 months), the trial was designed to provide at least 83% power (with a two-sided alpha = 0.05) to detect an 18% relative reduction in the composite primary endpoint (from 20% to 16.4%) in the invasive strategy compared with the conservative strategy. Enrollment began in mid-2012 and ended recently (January 31, 2018) with 5,179 participants randomized in 328 sites in 37 countries.^1^^,^^2^

## Modification of the Trial Protocol

Recently we learned that the protocol of the ISCHEMIA trial published on ClinicalTrials.gov had a major modification in January 2018, only 11 months before the estimated completion of this 7-year trial. The primary endpoint was changed from the composite of cardiovascular death or nonfatal MI (2012 version) to a 5-component endpoint that also includes resuscitated cardiac arrest, hospitalization for unstable angina and hospitalization for heart failure (January 2018 version).^1^^,^^2^

Then, in February 2018, the full version of the 2012 ISCHEMIA protocol was published on ClinicalTrials.gov. This version of the protocol stated that if the incidence of the primary endpoint pooled across randomized groups is lower than expected, an independent advisory panel can decide to extend the follow-up period or change the primary endpoint.

Strict adherence to study protocol is a cornerstone of the trial methodology. Extending follow-up improves precision towards the true effect and conserves the primary hypothesis of the trial. By contrast, the current wisdom in randomized clinical trials is that once the primary endpoint is selected, the trial should proceed with no further changes. Although trialists recognize that there may be appropriate reasons for modifying endpoints after the trial has started,^3^ evidence suggests that such changes often appear to favor the intervention group,^4^ raising the risk that failure to adhere to predetermined endpoints can inflate type I errors.

A change in primary endpoint is considered appropriate and unbiased when the decision is based on external information, independent of the trial data, such as the results of other studies. On the other hand, modification based on data from the trial itself will have a detrimental effect on the validity of the trial’s findings. A decision based on the pool incidence of events permits reasonable prediction of the main result and induces operational bias.^3^

## The Elephant in the Room

The decision to modify the primary endpoint of ISCHEMIA discards the original hypothesis and misses the elephant in the room: the incidence of death or MI was lower than expected, suggesting that the prognosis of patients with stable coronary disease is quite satisfactory. Such a good overall prognosis may reduce statistical power, but also makes futile the choice for an invasive procedure expected to protect patients from an unlikely outcome, at the expense of physical and mental stress, unintended consequences and monetary costs. In fact, the need for a higher than expected statistical power indicates the intention to detect an absolute risk reduction that may be clinically irrelevant.

The new components added to the composite primary endpoint also have implications for the trial’s findings. The original outcomes of cardiovascular death or MI are unequivocal, whereas hospitalization for angina or heart failure is mediated by a physician’s reaction to a clinical scenario. In an open study such as ISCHEMIA, it is possible that the knowledge that the patient was not revascularized could lower the physician's threshold for admitting patients due to symptoms.

Although unstable angina and MI belong to the same spectrum of pathophysiological processes collectively described as acute coronary syndromes, the diagnosis of unstable angina involves significant subjectivity on the part of the treating clinician, the investigator, and adjudication committees.^5^ Additionally, the prognostic relevance of unstable angina is much lower than that of MI and, of course, cardiovascular death. Therefore, the inclusion of hospitalization for unstable angina in the composite primary endpoint is susceptible to ascertainment bias and may alter the results towards a benefit for the routine invasive strategy.

Heart failure is a heterogeneous syndrome related not only to atherosclerosis but also to hypertension, renal disease, and other causes that are generally not discernible from the records available to the study adjudicators. It is also often difficult to distinguish heart failure from other causes of acute dyspnea.

In conclusion, it is highly questionable whether improving statistical power at the cost of impairing validity and relevance justifies this protocol modification. Changing the primary endpoint of a trial often evokes cynicism from the medical community and a study that uses a less relevant end point may not provide answers to clinically important questions. Time will tell whether such a strategy was a wise decision.
